# Therapeutics of Early Rising

**Published:** 1884-07

**Authors:** 


### The Therapeutics of Early Rising.

The subject of early rising has a physiological and a therapeutical aspect.

It may be laid down as a general proposition, applicable to the great majority of men, women, and children in health, that the proverb "early to bed, early to rise," etc., embodies sound common sense, and is safe to follow, enabling those who conform to it to perform the greatest amount of work of which they are capable with economy of time, of eyesight, of nervous energy, and of the general health.

The tendency of modern civilization has been to lead people greatly to disregard nature's law (to which the inferior animals generally conform), whereby night is the time for rest, and day is the time for toil. This law is the result of natural selection, as pointed out by one of its great apostles. An animal so constituted that waste and repair were balanced from moment to moment throughout the twenty-four hours would, other things equal, be overcome by an enemy or competitor that could evolve greater energy during the hours when light facilitates action at the expense of being less energetic during the hours of darkness and concealment.

Early rising is the proper sequel of early retiring, and when recommended as a hygienic rule it is with the presupposition that ample time—which with every individual is a fixed quantity—shall have been devoted to sleep.

The majority of laboring people, in accordance with the necessity in most adults for about eight hours' sleep a day, have wisely chosen the period between nine o'clock in the evening and early morning, and farmers who, retiring in the summer at dark, arise at daybreak, testify to the extraordinary salubrity of the morning air. The atmosphere then is cooler and more invigorating, more heavily charged with oxygen than, later in the day when rareified by the heat of the sun. There is no time so favorable for exercise, as students well know who are in the habit of taking long walks, or practicing gymnastics in the morning before breakfast. An hour spent in exercise at this time of day will fortify a student more than two hours of bodily training at midday, and this season should be chosen for physical rather than for mental exercises.

If sufficiency of sleep is necessary, excess of time spent in repose is enervating and harmful; it promotes languor of the circulation, and thus induces torpor and congestion of the viscera; it interferes with the energetic performance of the respiratory function, on which the due oxygenation of all the tissues depends; muscular tonicity and nerve nutrition are also enfeebled by prolonging the season of sleep.

From what has been above said it may be inferred that in pathological states characterized by deficient metabolism of nitrogenous waste elements, where, in common parlance, the biliary function is said to be deranged, early rising, with exercise in the open air, will be of powerful therapeutic efficacy. The blood is charged with the products of defective oxidation, the result of over-eating or over-work (or both combined), and all the symptoms of liver indigestion ensue. Here the best hygienic advice is that of old Dr. Abernethy, "An axe, a cord of wood, and five o'clock in the morning!" Under this regimen the "billiousness," with its attendant headaches, malaise, loss of appetite, etc., will often disappear as if by magic. If it is "the early bird" that catches "the worm," it is the early bird that can digest the worm. Dr. Abernethy's advice applies to many cases of simple dyspepsia, which will be more speedily and permanently benefitted by early rising than by wine bitters and pepsine; it may also be indicated in gout and diabetes, which are diseases of mal-nutrition.

As for nervous disorders, those characterized by spasm are, as a rule, more amenable to this kind of hygiene than paretic diseases, a recumbent posture increasing spinal reflex activity.

Epilepsy and hysteria have both been benefitted by the establishment of correct habits of sleep and by early rising, and to a certain class of debilitated young men (with whom every physician has much experience) no counsel is more important than to rise as soon as the first sound sleep is over. As for cases characterized by exhaustion and loss of power (neurasthenia and the paretic diseases of the nervous system), long-continued rest is often the only treatment that does any good. The damaged nervous elements have lost the power of rapid recuperation under sleep, and early rising would, in such patients, be a cruelty if not an impossibility. Reserve force is lacking, and morning weariness is often overpowering. Other persons are neurasthetic from want of a proper hygienic education, and cannot be too much exhorted to curtail the hours devoted to enervating inaction. To such patients the effort, persisted in, of early "springing from the bed of sloth," and engaging in some energetic occupation, is a victory over their infirmity.

In anæmia and chlorosis a "morning air bath," as Dr. Franklin styles it, is often more beneficial than a course of iron and arsenic.

The victim of insomnia must sometimes, perforce, lie abed in the morning; hours lost in vain attempts to go to sleep may be partially made up by prolonging the season of rest, and subsomnolent states are, as Dr. Crichton Browne observes, the next best thing to sleep. For the great majority, however, of people who are not bedridden, a matutinal walk is better than medicine.—*Boston Med. and Surg. Journal.*

